# Field Relevant Variation in Ambient Temperature Modifies Density-Dependent Establishment of *Plasmodium falciparum* Gametocytes in Mosquitoes

**DOI:** 10.3389/fmicb.2019.02651

**Published:** 2019-11-15

**Authors:** Ashutosh K. Pathak, Justine C. Shiau, Matthew B. Thomas, Courtney C. Murdock

**Affiliations:** ^1^Department of Infectious Diseases, College of Veterinary Medicine, University of Georgia, Athens, GA, United States; ^2^Center for Ecology of Infectious Diseases, University of Georgia, Athens, GA, United States; ^3^Center for Tropical Emerging Global Diseases, University of Georgia, Athens, GA, United States; ^4^The Department of Entomology, Center for Infectious Disease Dynamics, The Pennsylvania State University, University Park, PA, United States; ^5^Odum School of Ecology, University of Georgia, Athens, GA, United States; ^6^Center for Vaccines and Immunology, University of Georgia, Athens, GA, United States; ^7^Riverbasin Center, University of Georgia, Athens, GA, United States

**Keywords:** malaria transmission, diurnal temperature range, gametocytemia, vector competence, oocysts, sporozoites, parasite aggregation, human infectious reservoir

## Abstract

The relationship between *Plasmodium falciparum* gametocyte density and infections in mosquitoes is central to understanding the rates of transmission with important implications for control. Here, we determined whether field relevant variation in environmental temperature could also modulate this relationship. *Anopheles stephensi* were challenged with three densities of *P. falciparum* gametocytes spanning a ~10-fold gradient, and housed under diurnal/daily temperature range (“DTR”) of 9°C (+5°C and −4°C) around means of 20, 24, and 28°C. Vector competence was quantified as the proportion of mosquitoes infected with oocysts in the midguts (oocyst rates) or infectious with sporozoites in the salivary glands (sporozoite rates) at peak periods of infection for each temperature to account for the differences in development rates. In addition, oocyst intensities were also recorded from infected midguts and the overall study replicated across three separate parasite cultures and mosquito cohorts. While vector competence was similar at 20 DTR 9°C and 24 DTR 9°C, oocyst and sporozoite rates were also comparable, with evidence, surprisingly, for higher vector competence in mosquitoes challenged with intermediate gametocyte densities. For the same gametocyte densities however, severe reductions in the sporozoite rates was accompanied by a significant decline in overall vector competence at 28 DTR 9°C, with gametocyte density *per se* showing a positive and linear effect at this temperature. Unlike vector competence, oocyst intensities decreased with increasing temperatures with a predominantly positive and linear association with gametocyte density, especially at 28 DTR 9°C. Oocyst intensities across individual infected midguts suggested temperature-specific differences in mosquito susceptibility/resistance: at 20 DTR 9°C and 24 DTR 9°C, dispersion (aggregation) increased in a density-dependent manner but not at 28 DTR 9°C where the distributions were consistently random. Limitations notwithstanding, our results suggest that variation in temperature could modify seasonal dynamics of infectious reservoirs with implications for the design and deployment of transmission-blocking vaccines/drugs.

## Introduction

Targeting transmission of *Plasmodium falciparum* gametocytes to their mosquito vectors is now more pertinent than ever in light of the recent resurgence in disease incidence in sub-Saharan Africa but also for future efforts toward eliminating malaria (World Health Organization., 2018). An improved understanding of the relationship between gametocyte density in the human host and rates of transmission to vectors will benefit efforts to target individuals/sub-groups with dis-proportionately higher contribution to transmission (the “infectious reservoir”) (Stone et al., 2015). It will also assist in the development and efficacy assessment of transmission-reducing interventions (Rabinovich et al., 2017).

Assays of the relationship between gametocyte density and infectivity to the vector generally comprise one or more laboratory-reared vector species fed directly on an infected host or via artificial membrane, with/without serum replacement, prior knowledge of gametocyte density, or genotype (Churcher et al., 2013; Stone et al., 2015; Bousema and Drakeley, 2017; Goncalves et al., 2017; Bradley et al., 2018; Grignard et al., 2018; Slater et al., 2019). Taken together, these studies suggest a saturating positive relationship between the density of gametocytes in a human host and the proportion of mosquitoes that become successfully infected. Thus, it is generally assumed that hosts with higher gametocyte density will infect more mosquitoes on average, which in turn shapes recommendations for interventions.

However, significant variability exists around this relationship. For instance, despite the positive relationship between gametocyte density and mosquito infections, there is substantial uncertainty around this relationship. For example, some low density (also referred to as asymptomatic/sub-patent/sub-microscopic) carriers can contribute similar mosquito infection rates in mosquitoes as some high-density (symptomatic/patent/microscopic) carriers (Slater et al., 2019). Further, the saturating nature of the relationship suggests that high-density carriers offer no additional benefits to transmission (Stone et al., 2015; Bradley et al., 2018), which is difficult to reconcile with, for example, the evidence that rates of gametocytogenesis are under such strong selection pressure in sub-Saharan Africa (Duffy et al., 2018; Rono et al., 2018; Usui et al., 2019).

The variation around this relationship is often attributed to variation in host immuno-physiology, different mosquito species/populations, and differences across parasite genotypes in gametocyte investment. Most of the studies investigating the relationship between gametocytemia and infectivity to mosquitoes have been performed in mosquitoes housed at a single, constant temperature (e.g., 26°C) (Bousema et al., 2012; Churcher et al., 2013; Stone et al., 2015, 2018; Bradley et al., 2018; Tadesse et al., 2018; Slater et al., 2019). However, within minutes of ingestion by a mosquito, the malaria parasite is subject to an environment marked by substantial variation in temperature throughout the day, across seasons, and geographic region (Paaijmans et al., 2010; Lahondere and Lazzari, 2012; Murdock et al., 2012, 2014, 2016; Mordecai et al., 2013; Johnson et al., 2015; Ryan et al., 2015; Sinden, 2015; Beck-Johnson et al., 2017). Temperature has strong non-linear effects on mosquito infection rates and overall vector competence (with permissive temperatures for transmission ranging from 20 to 30°C and a predicted thermal optimum of 25°C), and is one of the most reliable environmental predictors of both geographical distribution and seasonal transmission dynamics (Mordecai et al., 2013; Johnson et al., 2015; Reiner et al., 2015; Ryan et al., 2015; Murdock et al., 2016; Shah et al., 2019).

The current study provides proof-of-concept evidence showing how field relevant variation in daily temperatures modulate the interaction between gametocyte density and infectivity to the mosquito vector. Female *Anopheles stephensi* were challenged with three densities of *P. falciparum* NF54 spanning an order of magnitude and housed at thermal regimes pertinent to malaria transmission: daily temperature fluctuations of a total of 9°C (+5°C/−4°C) around mean temperatures of 20, 24, and 28°C, respectively (Paaijmans et al., 2010; Blanford et al., 2013; Murdock et al., 2016). Metrics of parasite infection were assessed as the proportion of mosquitoes with oocysts in the midguts and their corresponding burdens, in addition to the proportions of mosquitoes carrying sporozoites in the salivary glands.

## Materials and Methods

### Study Design

**Figure 1:**
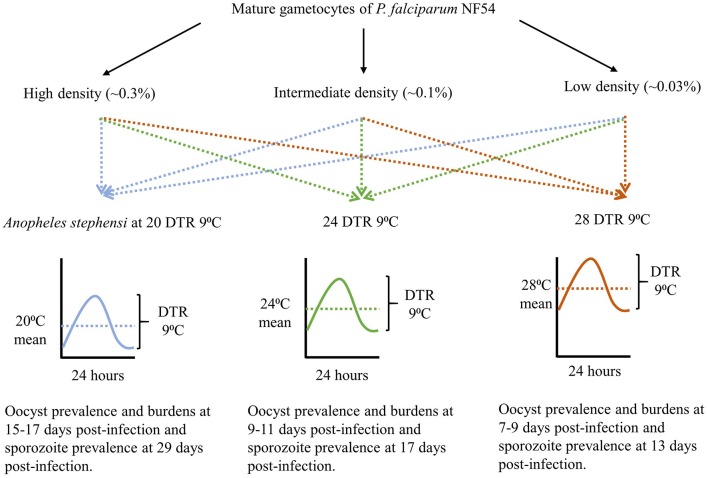
The study design showing gametocyte densities, temperature regimes, and time of sampling for each temperature, to account for the differences in development rates.

The overall study design is depicted in Figure 1 with the indicated temperature regimes adapted from a previous study (Murdock et al., 2016). Female *An. stephensi* from the same cohort were sorted into nine groups/cups with each temperature regime receiving three cups each, 24 h prior to the day of infection. On the day of infection, mature gametocytes of *P. falciparum* NF54 generated *in vitro* (Pathak et al., 2018) were serially diluted 3-fold with naïve RBCs to obtain blood-meals representing three final parasite densities with a 9-fold difference between the highest and lowest densities (~an order of magnitude). Aliquots of the three blood-meals with the varying gametocyte density were offered to the three cups, respectively, at each temperature regime. The experiments were replicated three times with independent mosquito cohorts and parasite cultures. Since the primary objective was to determine the effect of temperature on gametocyte density and vector competence, this experimental design ensured that within each replicate, all three temperature regimes would be assessed simultaneously with the same starting parasite and vector populations in order to reduce any unexpected variations over and above those attributable to temperature. Lastly, all mosquito infections were performed between 1800 and 1900 h which represents “dusk” on a circadian scale when Anopheline mosquitoes are most active (Rund et al., 2016).

All chemicals and consumables were purchased from Fisher Scientific Inc. unless stated otherwise.

### *In vitro* Parasite Cultures

Routine asexual cultures of *P. falciparum* NF54 and induction of gametocytogenesis *in vitro* were performed with cryo-preserved red blood cells (RBCs) as described in detail previously (Pathak et al., 2018). Gametocytogenesis of *P. falciparum* NF54 *in vitro* was monitored with Giemsa staining until 12 days post-culture, after which we assessed cultures for infectiousness through daily assays of male gametogenesis (Pathak et al., 2018). Giemsa stained slides were visualized at a 1000x magnification with an oil immersion lens while gametogenesis was assessed *in vitro* at 400x magnification under differential interference contrast setting on a Leica DM2500 upright microscope. Assays were performed on a 100 μl aliquot of culture re-suspended in fresh media. Infectiousness was determined in duplicate by quantifying male gametogenesis *in vitro* (ex-flagellation) with 10 μl culture volumes on a hemocytometer following incubation in a humidified chamber set to 24°C for 20 min. The remaining culture (~80 μl) was concentrated by centrifugation at 1,800 g for two min at room temperature and Giemsa stained smears prepared as described above.

### Mosquito Husbandry and Experimental Infections

*An. stephensi* colonies (Walter Reed Army Institute of Research, wild-type Indian strain) were housed in a walk-in environmental chamber (Percival Scientific, Perry, IA) at 27°C ± 0.5°C, 80% ± 5% relative humidity, and under a 12 h light: 12 h dark photo-period schedule. Adult mosquitoes were maintained on 5% dextrose (w/v) and 0.05% para-aminobenzoic acid (w/v) and provided whole human blood in glass-jacketed feeders (Chemglass Life Sciences, Vineland, NJ) through parafilm membrane maintained at 37°C to support egg production. Husbandry procedures followed methods outlined previously (Pathak et al., 2018). Briefly, eggs were rinsed twice with 1% house-hold bleach (v/v, final concentration of 0.06% sodium hypochlorite) before surface-sterilization for 1 min in the same solution at room temperature. Bleached eggs were washed with 4–5 changes of deionized water and transferred to clear plastic trays (34.6 cm L × 21.0 cm W × 12.4 cm H) containing 500 ml of deionized water and 2 medium pellets of Hikari Cichlid Gold fish food (Hikari USA, Hayward, CA) and allowed to hatch for 48 h. Hatched L1 larvae were dispensed into clear plastic trays (34.6 cm L × 21.0 cm W × 12.4 cm H) at a density of 300 larvae/1,000 ml water and provided the same diet until pupation. The feeding regime consisted of 2 medium pellets provided on the day of dispensing (day 0) followed by the provision of a further 2, 4, 4, and 4 medium pellets on days 4, 7, 8, and 9, respectively. This regime allows >85% larval survival and >90% pupation within 11 days with a sex ratio of 1:1 adult males and females (unpublished observations).

Mosquito infections were performed with ~100, 3, to 7 day old female, host-seeking *An. stephensi* sorted into nine 16/32 oz. soup cups. Three cups each were transferred to the respective temperature regimes and acclimated for ~24 h. On the day of infection, ex-flagellation was quantified from the cultures, in addition to gametocyte density from 3,000 to 5,000 RBCs stained with Giemsa, as described above. Parasite infected RBCs were collected into a pre-weighed 15 ml conical centrifuge tube and concentrated by centrifugation at 1800 × g for 2 min at low brake setting. The media supernatant was aspirated, and weight of packed, infected RBCs estimated after subtracting the weight of the empty tube. The infected RBC pellet was then resuspended in 3 volumes of a 33% hematocrit suspension of naïve, freshly washed RBCs in human serum to achieve a hematocrit of ~45–50%. This suspension was then serially diluted 3-fold a further two times by adding 2 volumes of naïve RBCs resuspended in human serum at 45–50% hematocrit to 1 volume of the preceding dilution resulting in three final concentrations of stage V gametocytemia (~0.3, 0.1, and 0.03%) used throughout this study. This dilution scheme was developed with the objective of achieving a mature gametocytemia of ~0.2–0.3% at the first dilution based on the gametocytemia recorded in the flasks on the day of infection and was representative of densities collated from independent studies (Adjalley et al., 2011; Miura et al., 2013, 2016; Stone et al., 2013; Eldering et al., 2017).

We then added an equal volume of each concentration of gametocytes to water-jacketed glass feeders maintained at ~37°C. All nine cups of mosquitoes were placed under the respective feeders and allowed to feed for 20 min. For estimating gametocyte density, smears were prepared from the blood-meal corresponding to the 1:2 dilution for Giemsa staining. Mosquitoes were returned to the respective temperature regimes and starved for a further 48 h to eliminate any partial or non-blood fed individuals after which they were provided cotton pads soaked in 5% dextrose (w/v) and 0.05% para-aminobenzoic acid (w/v) for the remainder of the study, as described previously (Pathak et al., 2018).

### Parasite Infection Measurements

Different metrics of parasite infection were estimated at time points corresponding to peak infection intensities specific to each temperature regime. Oocyst and sporozoite rates were measured as the proportion of mosquitoes with oocysts on their midguts or sporozoites in their salivary glands, respectively. Oocyst intensity, or the mean number oocysts per midgut, was used as a metric for overall parasite burden. Specifically, midguts and salivary glands were dissected to assess infection at the following times post-infection: 20 DTR 9°C, 15–17 days post-infection for oocysts and 29 days post-infection for sporozoites; 24 DTR 9°C, 9–11 days post-infection for oocysts and 17 days post-infection for sporozoites; and 28 DTR 9°C, 7–9 days post-infection for oocysts and 13 days post-infection for sporozoites. At each time point, ~25–30 mosquitoes were vacuum aspirated directly into 70% ethanol and vector competence measured as described previously (Pathak et al., 2018). Briefly, midguts were dissected, and oocysts enumerated at 400× magnification with a Leica DM2500 under DIC optics. For sporozoite rates, salivary glands were dissected into 5 μl of PBS, ruptured by overlaying a 22 mm^2^ coverslip and checking for presence/absence of sporozoites at either 100× or 400× magnification with the same microscope.

### Data Analyses

All data analyses were performed in RStudio (Version 1.1.463), an integrated development environment for the open-source R package (Version 3.5.2) (RSTUDIO Team, 2016; R Core Team, 2018). Graphical analyses were performed with the “ggplot2” package (Wickham, 2016). Vector competence was statistically modeled using Generalized linear mixed-effects models (GLMMs) with the choice of family/distribution based on the dependent variable—(1) Oocyst and sporozoite rates were modeled as the probability of being infected and infectious, respectively, using a beta-binomial distribution (family = beta-binomial, link = “logit”), and (2) oocyst intensity with a negative binomial distribution (family = nbinom2, link = “log”) with the “glmmTMB” package (Brooks et al., 2017). Predictors/fixed effects comprised temperature, gametocyte density and in the case of rates, site of infection, i.e., midguts or salivary glands, with the relationships modeled up to three-way interactions. Temperature and site of infection were classified as categorical fixed effects while density was specified as a continuous variable exerting a linear (*x*) and quadratic (*x*^2^) effect on the dependent variables (Crawley, 2013). Since technical constraints meant reliable parasite counts were only available for the highest parasite concentrations (1:2 dilution), dilution was used as a proxy for gametocyte density based on the fact that all three biological replicates were performed with the same series of dilutions of the original parasite culture (1:2, 1:6, and 1:18). For all models, the random effect structure allowed for variation in the intercepts between biological replicates and/or between temperatures nested within each replicate.

The choice of family for modeling oocyst intensity was based on likelihood-based information criteria as recommended in the “bbmle” package (Bolker and R Development Core Team, 2017), dispersion characteristics of residuals using the “DHARMa” package (Hartig, 2019), and where possible, the co-efficient of determination (“Pseudo-R-squared”) using the “sjstats” package (Lüdecke, 2018). Tests for overdispersion were performed using a predetermined threshold ratio of squared Pearson residuals over the residual degrees of freedom (overdispersion ratio = <1.5) and a Chi-squared distribution of the squared Pearson residuals with *p* > 0.05, as described previously (Pathak et al., 2018). Once overdispersion was accounted for, the marginal means estimated by each model were then used to perform pairwise comparisons between parasite densities nested within each temperature regime, using Tukey's contrast methods in the “emmeans” package and adjusting for multiple comparisons (Lenth, 2019).

## Results

### The Effect of Temperature and Gametocyte Density on Oocyst and Sporozoite Rates

**Figure 2:**
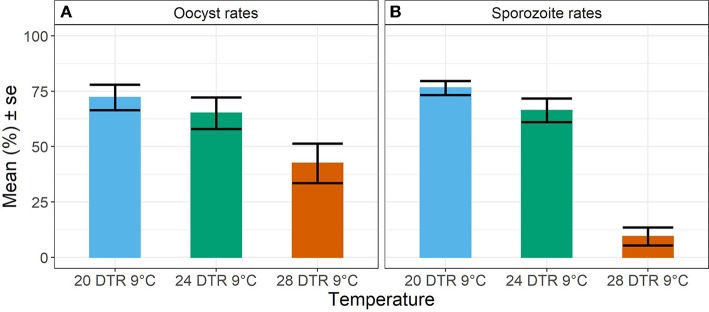
Oocyst rates in midguts **(A)** and sporozoite rates in salivary glands **(B)** at the three temperature regimes. Values represents mean ± standard error (se) of prevalence in percent units from three biological replicates.

Overall, we observed a main effect of temperature on both oocyst and sporozoite rates. Oocyst and sporozoite rates decreased with increasing temperature, with the most notable decline at 28 DTR 9°C (Log-Odds = −1.505, standard error (se) = 0.328, z-value = −4.59, *p* < 0.001) (Figure 2, Table 1). Sporozoite rates largely mirrored the oocyst rates at the two cooler temperatures but not at 28 DTR 9°C where sporozoite establishment declined significantly (Log-Odds = −2.446, se = 0.561, z-value = −4.362, *p* < 0.001) (Figure 2, Table 1). A non-linear quadratic (“hump-shaped”) relationship was noted between gametocyte density and overall vector competence (Log-Odds = −3.72, se = 1.45, z-value = −2.566, *p* = 0.01), likely driven by the two cooler temperatures where oocyst rates were highest at intermediate gametocyte densities (Figure 3, Table 1, and Supplementary Table 1). At the warmest temperature of 28 DTR 9°C, gametocyte density showed positive, linear effects on both oocyst and sporozoite rates (Log-Odds = 4.099, se = 2.056, z-value = 1.993, *p* = 0.046) (Figure 3, Table 1, and Supplementary Table 1). Overall, the model was able to explain 44.6% of the variation in the data with the predictors accounting for 42.7% of this fit. Pairwise comparisons of marginal means estimated by the models suggested clear differences between gametocyte density and oocyst rates at 28 DTR 9°C, but not at the two cooler temperatures where the proportion of mosquitoes infected with oocysts was marginally higher at the intermediate relative to the lowest gametocyte densities (Supplementary Table 2).

**Table 1:** Statistical models for prevalence of oocysts and sporozoites in midguts and salivary glands respectively.

**Predictors (reference group = 20 DTR 9^**°**^C)**	**Log-odds**	**std. error**	**Z-value**	***p***
(Intercept)	1.065	0.281	3.785	**<0.001**
24 DTR 9°C	−0.359	0.309	−1.162	0.245
28 DTR 9°C	−1.505	0.328	−4.590	**<0.001**
Sporozoite prevalence (vs. oocyst prevalence)	0.148	0.332	0.447	0.655
Gametocyte density (linear trend)	2.116	1.383	1.530	0.126
Gametocyte density (quadratic/“hump-shaped” trend)	−3.720	1.450	−2.566	**0.010**
24 DTR 9°C * sporozoite prevalence (vs. oocyst prevalence)	−0.160	0.430	−0.372	0.710
28 DTR 9°C * sporozoite prevalence (vs. oocyst prevalence)	−2.446	0.561	−4.362	**<0.001**
24 DTR 9°C * Gametocyte density (linear trend)	−1.022	1.854	−0.551	0.582
28 DTR 9°C * Gametocyte density (linear trend)	4.099	2.056	1.993	**0.046**
24 DTR 9°C * Gametocyte density (quadratic/“hump-shaped” trend)	0.256	1.963	0.130	0.896
28 DTR 9°C * Gametocyte density (quadratic/“hump-shaped” trend)	−0.047	2.114	−0.022	0.982
Sporozoite prevalence * Gametocyte density (linear trend)	0.654	2.270	0.288	0.773
Sporozoite prevalence * Gametocyte density (quadratic/“hump-shaped” trend)	3.396	2.173	1.563	0.118
24 DTR 9°C * sporozoite prevalence * Gametocyte density (linear trend)	0.054	2.983	0.018	0.986
28 DTR 9°C * sporozoite prevalence * Gametocyte density (linear trend)	−0.723	3.878	−0.186	0.852
24 DTR 9°C * sporozoite prevalence * Gametocyte density (quadratic/“hump-shaped” trend)	0.451	2.921	0.154	0.877
28 DTR 9°C * sporozoite prevalence * Gametocyte density (quadratic/“hump-shaped” trend)	−3.374	3.954	−0.853	0.394
**Random effects**				
Random variation in intercepts between temperatures nested within each biological replicate	0.03			
Random variation in intercepts between the three biological replicates	0.08			
Number of observations (i.e., mosquitoes sampled)	1469^#^			
Marginal *R*^2^/Conditional *R*^2^	0.427/0.446			

# *For one biological replicate, sporozoite prevalence data for all three densities at 20 DTR 9°C and lowest density at 24 DTR 9°C was not available. Bolded values indicate statistically clear effects based on p < 0.05*.

**Figure 3:**
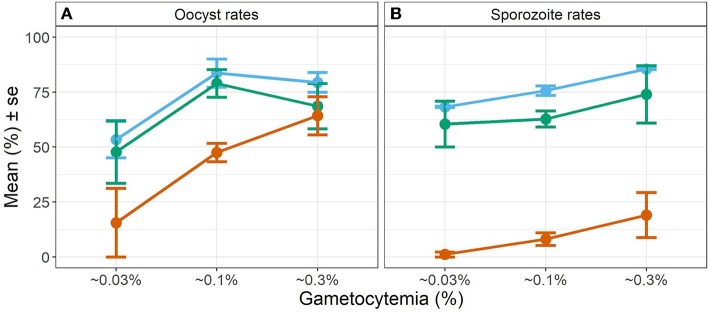
Effect of gametocyte density and temperature on oocyst rates in midguts **(A)** and sporozoite rates in the salivary glands **(B)**. Values represents means ± standard error (se) from three biological replicates. The color scheme is continued from Figure 2 with blue representing 20 DTR 9°C, green representing 24 DTR 9°C and red/vermillion indicating 28 DTR 9°C.

### The Effects of Temperature and Gametocyte Density on Oocyst Intensity

**Figure 4:**
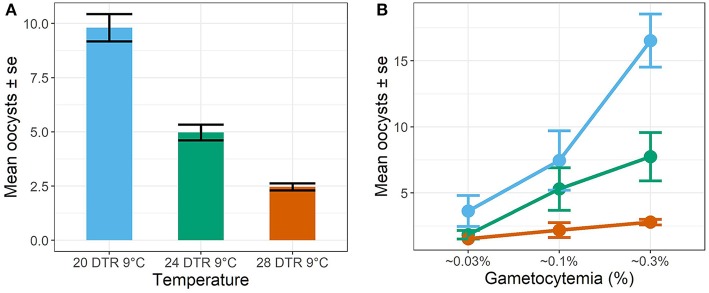
Oocyst intensities (midguts with oocysts ≥1, i.e., infected midguts) across temperatures **(A)** and gametocyte density **(B)**. Values represents means ± standard error (se) from three biological replicates.

In general, the mean number of oocysts per infected mosquito midgut showed significant declines as temperatures warmed, with intensities declining significantly at 24 DTR 9°C (Log-Mean = −0.58, se = 0.168, z-value = −3.449, *p* < 0.001) and especially at 28 DTR 9°C (Log-Mean = −1.386, se = 0.185, z-value = −7.481, *p* = 0.001) (Figure 4A). Further, increases in gametocytemia resulted in positive and linear increases in the mean number of oocysts per midgut (Log-Mean = −12.513, se = 1.078, z-value = 11.612, *p* < 0.001) (Figure 4B, Table 2, and Supplementary Figure 1), with weak evidence for a quadratic relationship with highest oocyst intensities at intermediate gametocyte densities (Log-Mean = −3.234, se = 1.072, z-value = −3.016, *p* = 0.003) (Figure 4B, Table 2, and Supplementary Table 3). Of the three temperatures, only the warmest temperature (28 DTR 9°C) showed a positive, linear relationship between gametocyte density and oocyst intensity (Log-Mean = −6.434, se = 2. 432, z-value = −2.747, *p* = 0.006) (Figure 4B, Table 2). Overall, the model was able to predict a total of 57.9% of the variation, with the predictors contributing 52.2% (Table 2). Pairwise comparisons of oocyst intensity suggest clear differences in the mean number of oocysts per midgut across all three gametocyte densities at the two cooler temperatures of 20 and 24 DTR 9°C (Supplementary Table 4). At 28 DTR 9°C, only the highest and lowest densities differed significantly in their contribution to burdens, however, this interpretation should be taken with caution since mosquitoes in two of the three experimental replicates at this temperature showed no evidence of infection at the lowest density, which may in turn have affected the pairwise comparisons.

**Table 2:** Statistical models for oocyst intensity (infected midguts).

	**Oocyst intensity**			
**Predictors (reference group = 20 DTR 9^**°**^C)**	**Log-mean**	**std. error**	**Z-value**	***p***
(Intercept)	2.112	0.143	14.772	**<0.001**
24 DTR 9°C	−0.580	0.168	−3.449	**0.001**
28 DTR 9°C	−1.386	0.185	−7.481	**<0.001**
Gametocyte density (linear trend)	12.513	1.078	11.612	**<0.001**
Gametocyte density (quadratic/“hump-shaped” trend)	−3.234	1.072	−3.016	**0.003**
24 DTR 9°C/Gametocyte density (linear trend)	−2.271	1.634	−1.390	0.165
28 DTR 9°C/Gametocyte density (linear trend)	−6.434	2.342	−2.747	**0.006**
24 DTR 9°C/Gametocyte density (quadratic/“hump-shaped” trend)	−2.749	1.624	−1.692	0.091
28 DTR 9°C/Gametocyte density (quadratic/“hump-shaped” trend)	−0.565	2.558	−0.221	0.825
**Random effects**				
Random variation in intercepts between temperatures within each replicate	0.03			
Random variation in intercepts between replicates	0.1			
Observations	504			
Marginal *R*^2^/Conditional *R*^2^	0.522/0.579			
GLMM family (“link”)	Negative binomial with variance increasing quadratically with the mean [glmmTMB family = nbinom2 (link = “log”)]			

*Bolded values indicate statistically clear effects based on p < 0.05*.

### The Effects of Temperature and Gametocytemia on the Distribution of Parasites Across Mosquitoes

**Figure 5:**
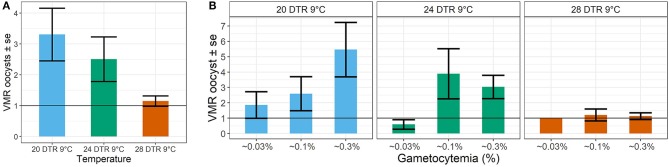
Effect of temperature **(A)** and gametocyte density **(B)** on the distribution of oocyst burdens across individual (infected) midguts at the three temperatures using the Variance to Mean Ratios (VMR) as the measure of dispersion (*s*^2^*: m*). Values represents means ± standard error (se) of the VMR from three biological replicates.

Simple phenomenological analyses of the oocyst distribution across individual mosquito midguts suggests strong gametocyte density- and temperature-dependent patterns (Figure 5 and Supplementary Figures 1, 2). To describe how parasite burdens are distributed across individual mosquito midguts, we quantified the variance to mean ratios (VMR). A VMR of ~1 indicates a random distribution of oocysts across dissected mosquito midguts while ratios > 1 indicate an uneven or aggregated oocyst distribution, with most midguts exhibiting few to no oocysts and a few midguts displaying high oocyst burdens (Wilson et al., 2002). In general, aggregation decreased with increases in temperature, with oocysts almost randomly distributed at the warmest temperature of 28 DTR 9°C regardless of initial gametocyte density. At the two cooler temperatures, aggregation increased with increasing gametocyte density, with this pattern most pronounced at the coldest temperature of 20 DTR 9°C.

## Discussion

Increasing temperatures were detrimental to *P. falciparum* fitness with declines in the mean proportion of mosquitoes infected with oocysts (oocyst rates) and infectious with sporozoites (sporozoite rates), as well as mean oocyst intensities. The main effects of temperature observed in this study fall in line with predicted temperature-vector competence relationships for malaria outlined in previous studies (Mordecai et al., 2013; Johnson et al., 2015; Shapiro et al., 2017). Oocyst and sporozoite rates were similar at the cooler temperatures of 20 and 24 DTR9°C but not at the warmest temperature of 28 DTR 9°C where sporozoite rates declined significantly. We also found oocyst intensity to decrease with increasing temperature. Taken together, our results suggest that the overall efficiency of malaria infection declines at the warmest temperature either due to a direct effect of temperature on parasite sporogony and/or an indirect effect mediated by mosquito immunological or physiological changes.

Interestingly, oocyst rates were comparable across temperatures at the highest gametocyte densities, despite the qualitative differences in the shape of the relationship at each temperature. For example, while the highest oocyst rates were achieved with intermediate gametocyte densities at the two cooler temperatures of 20 and 24 DTR 9°C, high gametocyte densities were required to achieve similar oocyst rates at 28 DTR 9°C and to ensure progression to the salivary glands. At the highest temperature, it is likely that the thermal stress was simply not conducive to sporozoite survival, sporozoite migration, and/or residence in the salivary glands (Mordecai et al., 2013; Johnson et al., 2015; Shapiro et al., 2017). Indeed, while parasite genotypes are likely to vary in their susceptiblity to high temperatures (Noden et al., 1995), our results appear to contrast with a previous study (Murdock et al., 2016), where vector competence was observed with the same parasite-mosquito combination at temperatures as high as 33°C. However, the gametocyte density offered to the mosquitoes was considerably higher (~0.8% and unpublished observations) than the highest density from the current study (0.3%) and suggests that this parasite genotype can infect successfully at high temperatures, provided gametocyte densities are high. Additionally, the same study showed similar oocyst and sporozoite rates (~55%) at 27 DTR 9°C, which corroborates our findings at 28 DTR9°C where high densities promoted parasite progression to the salivary glands. Taken together, the former study complements our current findings and reinforces the notion that variation in environmental temperature alters the relationship between gametocyte density and infection rates in mosquitoes.

In contrast to the effect of gametocyte density on oocyst rates, oocyst intensity in infected midguts showed a generally linear, positive relationship across all temperatures. These distinct midgut responses are in line with other work showing how oocyst burdens rarely saturate with increasing gametocyte density (Eldering et al., 2017; Bradley et al., 2018). However, our results also suggest that temperature can dramatically alter the effects of gametocyte density on the distribution of oocyst burdens across infected midguts. At the gametocyte densities tested here, oocyst distributions showed increased aggregation as gametocyte density increased at the two cooler temperatures, but not at the highest temperature where oocysts were randomly distributed regardless of gametocyte density. These results suggest increased gametocytemia at temperatures permissive for mosquito lifespan and parasite infection rates (Mordecai et al., 2013; Johnson et al., 2015; Shapiro et al., 2017) may increase competition among parasites. Competition can either manifest directly in response to limited host resources or indirectly due to increased host immune responses (Lefevre et al., 2017) resulting in high oocyst burdens in a few, highly nourished or susceptible hosts. In contrast, temperatures above the predicted thermal optimum for vector competence (Mordecai et al., 2013; Johnson et al., 2015; Shapiro et al., 2017) may have direct negative effects on parasite survival resulting in lower infection rates overall, less parasite competition within host, and more random distributions of parasites across hosts.

Our results have several implications for understanding malaria transmission. Identifying human infectious reservoirs and evaluating their contribution to overall transmission for targeted intervention will likely need to consider environmental context. For instance, low-density individuals might contribute as much as high-density carriers in regions of the world or times of season when temperatures are cooler and any intervention may therefore need to cover a relatively larger proportion of the host population. Likewise, the infectious reservoir may shift to high-density carriers in warmer regions of the world or times of season where, in principle, targeting high-density carriers may bring significant returns. Further, if ambient temperature modifies the outcomes of the relationship between gametocyte density and mosquito infection rates as shown here, it could provide an additional rationale for the seasonal and geographical distributions of allelic variants (*gdv1* and/or *AP2-g*), both of which are distinguished primarily by their differential investment in gametocytogenesis (Gadalla et al., 2016; Duffy et al., 2018; Rono et al., 2018; Usui et al., 2019). Finally, while higher temperatures could select for transmission of genotypes capable of higher gametocytogenesis, at lower temperatures the same genotype could be outcompeted due to density-dependent effects (Pollitt et al., 2013).

The interactions between temperature and gametocyte density described here also have several implications for current transmission reducing/blocking interventions. Transmission reducing interventions, such as the anti-gametocidal drug primaquine, could be much more effective in reducing transmission of artemisinin resistant parasites in warmer environments or times of season than in cooler seasons or geographic regions. Further, thermal variation in the field could have implications for current vaccine design and testing pipelines where the rate of reduction in oocyst intensity is the primary method of evaluation (Bompard et al., 2017). For instance, more effective antibody responses may be required at lower temperatures where oocyst intensity and sporozoite rates are highest. Finally, if transmission blocking vaccines are imperfect as early evidence suggests (Sagara et al., 2018), intermediate gametocytemia in some environmental contexts might actually boost oocyst rates: whether this enhancement will be reflected in subsequent transmission rates will ultimately depend on sporozoite rates, which appear to be less influenced by gametocyte density.

While our proof of concept study has several implications for understanding malaria transmission and control, there are several study limitations that may warrant consideration. *First*, it is likely that the gametocyte densities tested here are more in line with laboratory rather than field-based studies (Koepfli and Yan, 2018). Further, standard membrane feeding assays (SMFAs) are less efficient than direct membrane feeding assays (DMFAs) or direct skin-feeding assays (DFAs) (Bousema et al., 2012). We do not anticipate these effects will change the main result that temperature alters the gametocytemia-infection rate relationship. *Second*, this study was conducted using a “non-native” vector species-parasite genotype combination (Molina-Cruz et al., 2015). However, the effects of temperature on vector competence in *An. gambiae* is correlated with *An. stephensi* (Murdock et al., 2016; Eldering et al., 2017), and the study could prove to be timely considering the recent infestation of *An. stephensi* in cities of Eastern Africa (Faulde et al., 2014; Carter et al., 2018) and contribution to *P. falciparum* transmission (Seyfarth et al., 2019). *Third*, we did not measure sporozoite burdens in the salivary glands, and it is likely that higher temperatures and/or low oocyst intensities may result in reduced sporozoite numbers in the salivary glands (Stone et al., 2013; Miura et al., 2019) with potential implications for transmission to the human host (Churcher et al., 2017). Further, previous work in rodent malaria systems have demonstrated negative relationships between oocyst burdens and the number of sporozoites produced per oocyst (Pollitt et al., 2013; Moller-Jacobs et al., 2014) suggesting density-dependent effects might also influence sporozoite intensity. *Finally*, it is possible that mosquitoes seek particular microclimates, which could mitigate the effects of temperature on this relationship. However, there is limited evidence suggesting this is the case currently (Blanford et al., 2009), and much more work is needed to address this potential limitation.

In this study, we demonstrate that field relevant variation in ambient temperature alters the relationship between density of transmission stages and infection outcomes in the *An. stephensi*—*P. falciparum* system. We emphasize that the role of variation in field relevant factors in shaping this relationship is currently under appreciated, with potentially important implications for understanding malaria transmission and control. Of note, while only one regime was tested here (DTR 9°C), it is possible that the magnitude of fluctuations may result in different parasite fitness phenotypes and collectively argues for further investigating the relationship between gametocytemia and mosquito infection rates under field relevant conditions (Paaijmans et al., 2010; Blanford et al., 2013; Murdock et al., 2016). Further, due to the well-established effects of environmental variation like temperature on other aspects of vectorial capacity (e.g., mosquito mortality, biting rate, and the parasite extrinsic incubation periods), our studies highlight the need to look beyond the human infectious reservoir and mosquito midgut infection rates for characterizing local transmission.

## Data Availability Statement

The datasets generated for this study are available on request to the corresponding author.

## Author Contributions

AP designed the study, collected and analyzed the data, and wrote the manuscript. CM provided critical input on the study design, methods, data analysis, and manuscript preparation, as well as provided infrastructure and resources for the execution of the presented research. JS contributed to the mosquito husbandry and helped AP to collect data. MT provided comments and suggestions during the project and on the manuscript. All authors read and approved the final version of the manuscript.

### Conflict of Interest

The authors declare that the research was conducted in the absence of any commercial or financial relationships that could be construed as a potential conflict of interest.

## Supplementary Material

Supplementary Figure 1 Effect of gametocytemia on the distribution of oocyst burdens in infected midguts at the three temperature regimes depicted in the respective panes. Each box represents the interquartile range, with the line within each box indicating median oocyst intensities, bounded by the 25th and 75th percentile values. Error bars represent maximum and minimum values from the interquartile range, with the dots representing outliers. Data were aggregated from the three biological replicates.

Supplementary Figure 2 Effect of gametocytemia on the distribution of oocyst burdens (≥0) across all midguts (infected and un-infected) at 20 DTR 9°C (blue, **top** panes), 24 DTR 9°C (green, **middle** panes) and 28 DTR 9°C (red, **bottom** panes). Gametocyte densities are indicated in the strips above each plot. Values represent data from all 839 mosquito midguts from three biological replicates.

Supplementary Table 1 Statistical models for prevalence of oocysts and sporozoites in midguts and salivary glands for each temperature and DTR. ^#^ Sporozoite prevalence data from one biological replicate was not available. ^+^ Sporozoite prevalence data from the lowest density for one biological replicate was not available.

Supplementary Table 2 Pairwise comparisons of means predicted by the model in Table 1 in the main text for oocyst and sporozoite prevalence.

Supplementary Table 3 Statistical models for oocyst intensity (infected midguts) for each temperature.

Supplementary Table 4 Pairwise comparisons of means predicted by the model in Table 2 in the main text for oocyst abundance and intensity.
