# Supplementary figures and images for: Field Relevant Variation in Ambient Temperature Modifies Density-Dependent Establishment of Plasmodium falciparum Gametocytes in Mosquitoes
Source: Front Microbiol. 2019 Nov 15;10:2651. doi: 10.3389/fmicb.2019.02651 (PMC6873802; doi:10.3389/fmicb.2019.02651)

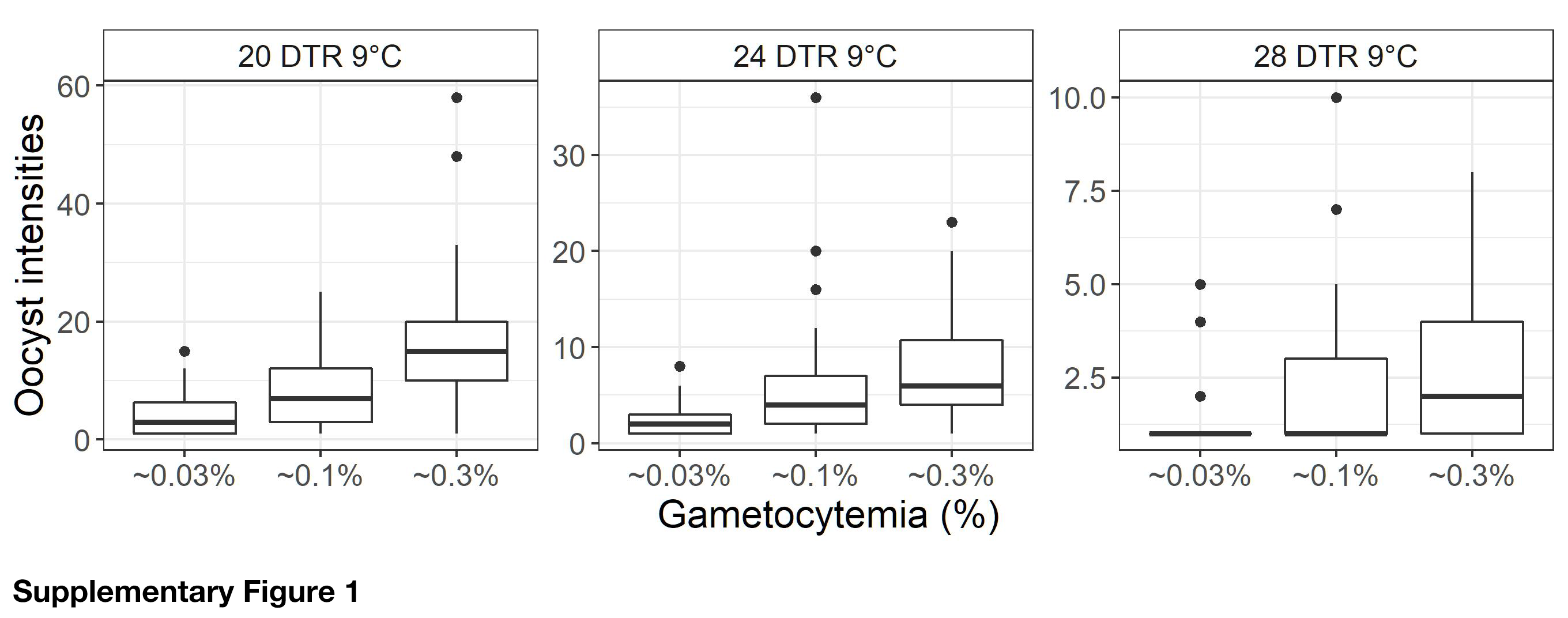

Supplement: Supplementary Figure 1 — Effect of gametocytemia on the distribution of oocyst burdens in infected midguts at the three temperature regimes depicted in the respective panes. Each box represents the interquartile range, with the line within each box indicating median oocyst intensities, bounded by the 25th and 75th percentile values. Error bars represent maximum and minimum values from the interquartile range, with the dots representing outliers. Data were aggregated from the three biological replicates. [file Image_1.tif]

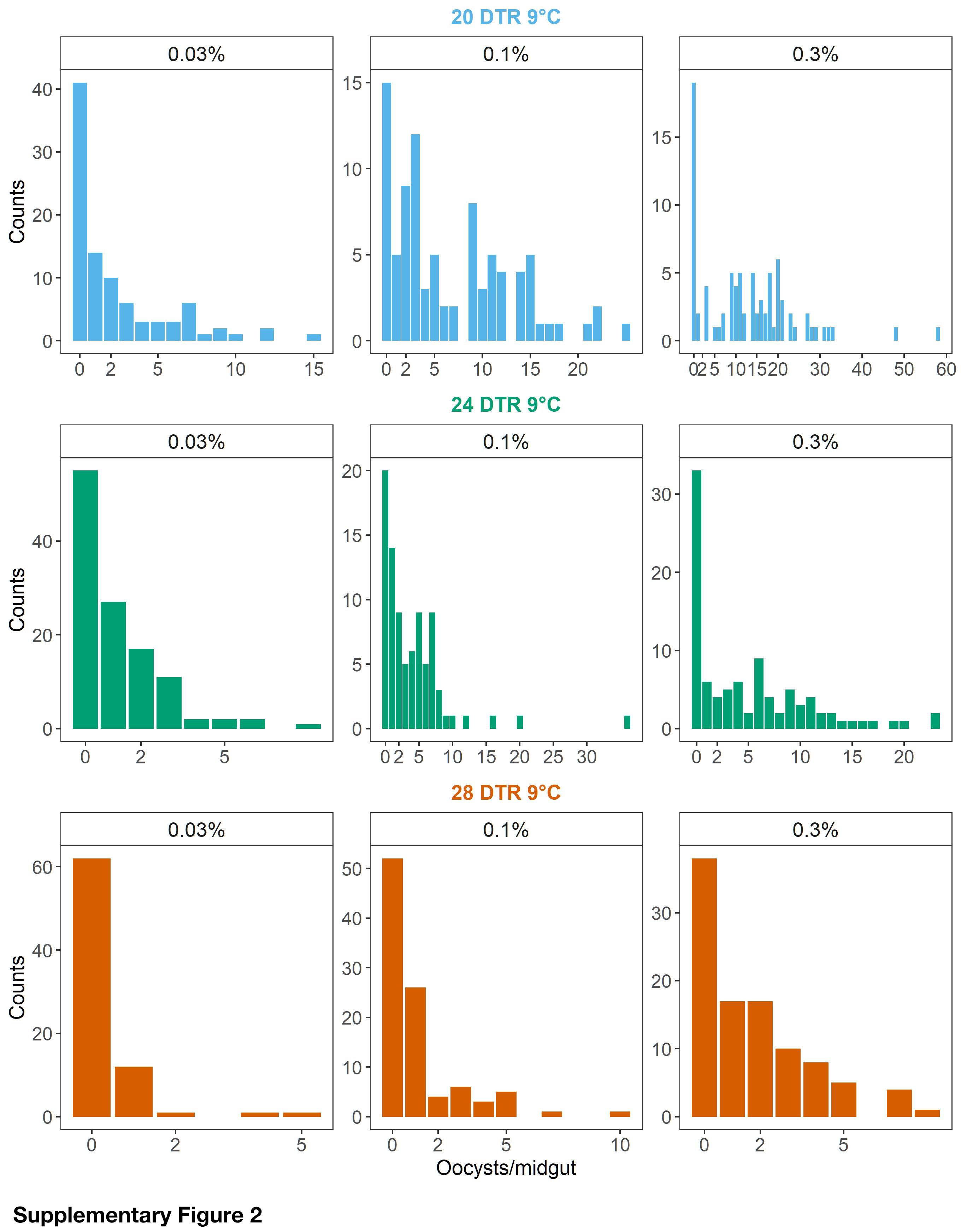

Supplement: Supplementary Figure 2 — Effect of gametocytemia on the distribution of oocyst burdens (≥0) across all midguts (infected and un-infected) at 20 DTR 9°C (blue, top panes), 24 DTR 9°C (green, middle panes) and 28 DTR 9°C (red, bottom panes). Gametocyte densities are indicated in the strips above each plot. Values represent data from all 839 mosquito midguts from three biological replicates. [file Image_2.tif]
