# Supplementary material for: Field Relevant Variation in Ambient Temperature Modifies Density-Dependent Establishment of Plasmodium falciparum Gametocytes in Mosquitoes
Source: Front Microbiol. 2019 Nov 15;10:2651. doi: 10.3389/fmicb.2019.02651 (PMC6873802; doi:10.3389/fmicb.2019.02651)
Supplement: Supplementary Table 1 — Statistical models for prevalence of oocysts and sporozoites in midguts and salivary glands for each temperature and DTR. # Sporozoite prevalence data from one biological replicate was not available. + Sporozoite prevalence data from the lowest density for one biological replicate was not available. [file Table_1.docx]

| **Supplementary table 1** | | | | | | | | | | | | |
| --- | --- | --- | --- | --- | --- | --- | --- | --- | --- | --- | --- | --- |
|  | **20 DTR 9⁰C** | | | | **24 DTR 9⁰C** | | | | **28 DTR 9⁰C** | | | |
| *Predictors* | *Log-Odds* | *std. Error* | *Z value* | *p* | *Log-Odds* | *std. Error* | *Z value* | *p* | *Log-Odds* | *std. Error* | *Z value* | *p* |
| (Intercept) | 1.021 | 0.146 | 6.995 | **<0.001** | 0.729 | 0.274 | 2.658 | **0.007** | -0.569 | 0.425 | -1.338 | 0.181 |
| Sporozoite prevalence | 0.199 | 0.232 | 0.858 | 0.390 | -0.02 | 0.282 | -0.073 | 0.942 | -2.04 | 0.575 | -3.547 | **<0.001** |
| Linear trend over parasite density | 1.07 | 0.551 | 1.942 | **0.052** | 0.558 | 0.75 | 0.743 | **0.457** | 4.241 | 1.287 | 3.293 | **<0.001** |
| Quadratic trend over parasite density | -1.947 | 0.579 | -3.363 | **<0.001** | -1.996 | 0.792 | -2.519 | **0.012** | -2.707 | 1.316 | -2.056 | **0.039** |
| Sporozoite prevalence * linear trend over parasite density | 0.53 | 0.926 | 0.573 | 0.566 | 0.483 | 1.161 | 0.416 | 0.677 | -1.015 | 2.268 | -0.447 | 0.654 |
| Sporozoite prevalence * quadratic trend over parasite density | 1.771 | 0.875 | 2.024 | **0.042** | 2.253 | 1.164 | 1.935 | **0.052** | 0.501 | 2.258 | 0.222 | 0.824 |
| **Random Effects** | | | | | | | | | | | | |
| Random variation in intercepts between the three biological replicates | 0.00 | | | | 0.15 | | | | 0.26 | | | |
| Number of mosquitoes sampled | 449^#^ | | | | 483^+^ | | | | 537 | | | |
