# Supplementary material for: Field Relevant Variation in Ambient Temperature Modifies Density-Dependent Establishment of Plasmodium falciparum Gametocytes in Mosquitoes
Source: Front Microbiol. 2019 Nov 15;10:2651. doi: 10.3389/fmicb.2019.02651 (PMC6873802; doi:10.3389/fmicb.2019.02651)
Supplement: Supplementary Table 2 — Pairwise comparisons of means predicted by the model in Table 1 in the main text for oocyst and sporozoite prevalence. [file Table_2.docx]

**Supplementary table 2**

| Oocyst prevalence (midguts) | | | | |
| --- | --- | --- | --- | --- |
| Temperature | **Pairwise comparisons**  **Gametocytemia 1 vs gametocytemia 2** | **estimate (log odds)** | **std. Error** | ***p*-value** |
| 20 DTR 9⁰C | ~0.03% vs. ~0.1% | -0.286 | 0.092 | **0.012** |
| 20 DTR 9⁰C | ~0.03% vs. ~0.3% | -0.233 | 0.095 | 0.053 |
| 20 DTR 9⁰C | ~0.1% vs. ~0.3% | 0.053 | 0.080 | 0.786 |
|  |  |  |  |  |
| 24 DTR 9⁰C | ~0.03% vs. ~0.1% | -0.277 | 0.093 | **0.015** |
| 24 DTR 9⁰C | ~0.03% vs. ~0.3% | -0.172 | 0.098 | 0.200 |
| 24 DTR 9⁰C | ~0.1% vs. ~0.3% | 0.105 | 0.090 | 0.477 |
|  |  |  |  |  |
| 28 DTR 9⁰C | ~0.03% vs. ~0.1% | -0.345 | 0.096 | **0.003** |
| 28 DTR 9⁰C | ~0.03% vs. ~0.3% | -0.507 | 0.093 | **<.0001** |
| 28 DTR 9⁰C | ~0.1% vs. ~0.3% | -0.162 | 0.100 | 0.249 |
|  |  |  |  |  |
| Sporozoite prevalence (salivary glands) | | | | |
| Temperature | **Pairwise comparisons**  **Gametocytemia 1 vs gametocytemia 2** | **estimate (log odds)** | **std. Error** | ***p*-value** |
| 20 DTR 9⁰C | ~0.03% vs. ~0.1% | -0.069 | 0.111 | 0.812 |
| 20 DTR 9⁰C | ~0.03% vs. ~0.3% | -0.165 | 0.104 | 0.266 |
| 20 DTR 9⁰C | ~0.1% vs. ~0.3% | -0.097 | 0.097 | 0.585 |
|  |  |  |  |  |
| 24 DTR 9⁰C | ~0.03% vs. ~0.1% | -0.005 | 0.120 | 0.999 |
| 24 DTR 9⁰C | ~0.03% vs. ~0.3% | -0.120 | 0.116 | 0.560 |
| 24 DTR 9⁰C | ~0.1% vs. ~0.3% | -0.116 | 0.102 | 0.503 |
|  |  |  |  |  |
| 28 DTR 9⁰C | ~0.03% vs. ~0.1% | -0.069 | 0.043 | 0.258 |
| 28 DTR 9⁰C | ~0.03% vs. ~0.3% | -0.134 | 0.059 | 0.074 |
| 28 DTR 9⁰C | ~0.1% vs. ~0.3% | -0.066 | 0.066 | 0.582 |
