# Supplementary material for: Field Relevant Variation in Ambient Temperature Modifies Density-Dependent Establishment of Plasmodium falciparum Gametocytes in Mosquitoes
Source: Front Microbiol. 2019 Nov 15;10:2651. doi: 10.3389/fmicb.2019.02651 (PMC6873802; doi:10.3389/fmicb.2019.02651)
Supplement: Supplementary Table 3 — Statistical models for oocyst intensity (infected midguts) for each temperature. [file Table_3.docx]

| **Supplementary table 3** | | | | | | | | | | | | |
| --- | --- | --- | --- | --- | --- | --- | --- | --- | --- | --- | --- | --- |
|  | **20 DTR 9⁰C** | | | | **24 DTR 9⁰C** | | | | **28 DTR 9⁰C** | | | |
| *Predictors* | *Log-Mean* | *Std. Error* | *Z-value* | *p* | *Log-Mean* | *Std. Error* | *Z-value* | *p* | *Log-Mean* | *Std. Error* | *Z-value* | *p* |
| (Intercept) | 2.065 | **0.183** | **11.242** | **<0.001** | 1.45 | **0.133** | **10.882** | **<0.001** | 0.848 | **0.106** | **7.953** | **<0.001** |
| Linear trend with gametocyte density | 7.7 | **0.687** | **11.199** | **<0.001** | 6.612 | **0.77** | **8.588** | **<0.001** | 2.197 | **0.759** | **2.895** | **0.003** |
| Quadratic trend with gametocyte density | -2.03 | .706 | -2.882 | **0.003** | -3.74 | **0.782** | **-4.777** | **<0.001** | -1.438 | 0.842 | -1.708 | 0.087 |
|  |  |  |  |  |  |  |  |  |  |  |  |  |
| **Random effects** |  |  |  |  |  |  |  |  |  |  |  |  |
| Random variation in intercepts between the three biological replicates | 0.09 | | | | 0.04 | | | | 0.02 | | | |
| Number of infected midguts | 191 | | | | 190 | | | | 123 | | | |
