# Supplementary material for: Field Relevant Variation in Ambient Temperature Modifies Density-Dependent Establishment of Plasmodium falciparum Gametocytes in Mosquitoes
Source: Front Microbiol. 2019 Nov 15;10:2651. doi: 10.3389/fmicb.2019.02651 (PMC6873802; doi:10.3389/fmicb.2019.02651)
Supplement: Supplementary Table 4 — Pairwise comparisons of means predicted by the model in Table 2 in the main text for oocyst abundance and intensity. [file Table_4.docx]

**Supplementary table 4**

| Oocyst intensity (infected midguts) | | | | |
| --- | --- | --- | --- | --- |
| Temperature | **Pairwise comparisons**  **Gametocytemia 1 vs gametocytemia 2** | **estimate (log odds)** | **std. Error** | ***p*-value** |
| 20 DTR 9⁰C | ~0.03% vs. ~0.1% | -3.681 | 0.83 | **<.0001** |
| 20 DTR 9⁰C | ~0.03% vs. ~0.3% | -12.253 | 2.049 | **<.0001** |
| 20 DTR 9⁰C | ~0.1% vs. ~0.3% | -8.572 | 1.73 | **<.0001** |
|  |  |  |  |  |
| 24 DTR 9⁰C | ~0.03% vs. ~0.1% | -3.07 | 0.637 | **<.0001** |
| 24 DTR 9⁰C | ~0.03% vs. ~0.3% | -5.61 | 1.009 | **<.0001** |
| 24 DTR 9⁰C | ~0.1% vs. ~0.3% | -2.54 | 0.838 | **0.0072** |
|  |  |  |  |  |
| 28 DTR 9⁰C | ~0.03% vs. ~0.1% | -0.975 | 0.44 | 0.0694 |
| 28 DTR 9⁰C | ~0.03% vs. ~0.3% | -1.533 | 0.475 | **0.0038** |
| 28 DTR 9⁰C | ~0.1% vs. ~0.3% | -0.558 | 0.403 | 0.3502 |
